# *catena*-Poly[{μ-*N*′-[2-(carboxylatomethoxy)benzylidene]-2-hydroxybenzohydrazidato}(methanol-κ*O*)nickel(II)]

**DOI:** 10.1107/S1600536813024161

**Published:** 2013-08-31

**Authors:** Feihua Luo, Huirong Du, Li Yang, Ping Zhang

**Affiliations:** a Department of Chemistry and Chemical Engineering, Sichuan University of Arts and Science, Sichuan Key Laboratory of Characteristic Plant Development Research, Dazhou, Sichuan 635000, People’s Republic of China; b Department of Chemistry and Chemical Engineering, Sichuan University of Arts and Science, Dazhou, Sichuan 635000, People’s Republic of China

## Abstract

In the title compound, [Ni(C_16_H_12_N_2_O_5_)(CH_3_OH)]_*n*_, the unique Ni^II^ ion is coordinated in a distorted octa­hedral environment by three O atoms and one N atom from a symmetry-unique ligand in the equatorial sites. Coordination of the axial sites is provided by an O atom of a symmetry-unique methanol ligand and an O atom of a carboxyl­ate group from a symmetry-related ligand, thus generating a one-dimensional polymer parallel to [010]. In the crystal, O—H⋯N hydrogen bonds and π–π inter­actions, with a centroid–centroid distance of 3.693 (2) Å, form a two-dimensional network parallel to (100). In addition, weak C—H⋯O and C—H⋯N hydrogen bonds complete a three-dimensional network. An intra­molecular O—H⋯O hydrogen bond is also observed.

## Related literature

For background information on nickel(II) carboxyl­ate compounds, see: Lu *et al.* (2010 ▶). For general information on the structures of carboxyl­ate and hydrazone compounds, see: Wu *et al.* (2007 ▶); Luo *et al.* (2010 ▶). 
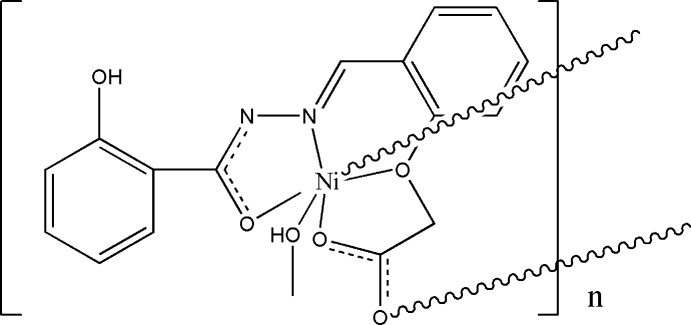


## Experimental

### 

#### Crystal data

[Ni(C_16_H_12_N_2_O_5_)(CH_4_O)]
*M* *_r_* = 403.03
Monoclinic, 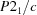

*a* = 10.0877 (14) Å
*b* = 8.1922 (10) Å
*c* = 20.570 (2) Å
β = 102.589 (8)°
*V* = 1659.0 (4) Å^3^
*Z* = 4
Mo *K*α radiation
μ = 1.21 mm^−1^
*T* = 296 K
0.20 × 0.20 × 0.15 mm

#### Data collection

Bruker SMART CCD diffractometer
12305 measured reflections
2921 independent reflections
2635 reflections with *I* > 2σ(*I*)
*R*_int_ = 0.027

#### Refinement

*R*[*F* ^2^ > 2σ(*F* ^2^)] = 0.029
*wR*(*F* ^2^) = 0.073
*S* = 1.08
2921 reflections
248 parameters
H atoms treated by a mixture of independent and constrained refinement
Δρ_max_ = 0.35 e Å^−3^
Δρ_min_ = −0.24 e Å^−3^

### 

Data collection: *SMART* (Bruker, 2001 ▶); cell refinement: *SAINT* (Bruker, 2001 ▶); data reduction: *SAINT*; program(s) used to solve structure: *SHELXS97* (Sheldrick, 2008 ▶); program(s) used to refine structure: *SHELXL97* (Sheldrick, 2008 ▶); molecular graphics: *PLATON* (Spek, 2009 ▶); software used to prepare material for publication: *SHELXTL* (Sheldrick, 2008 ▶).

## Supplementary Material

Crystal structure: contains datablock(s) I, New_Global_Publ_Block. DOI: 10.1107/S1600536813024161/lh5646sup1.cif

Structure factors: contains datablock(s) I. DOI: 10.1107/S1600536813024161/lh5646Isup2.hkl

Additional supplementary materials:  crystallographic information; 3D view; checkCIF report

## Figures and Tables

**Table 1:** Hydrogen-bond geometry (Å, °)

*D*—H⋯*A*	*D*—H	H⋯*A*	*D*⋯*A*	*D*—H⋯*A*
C7—H7⋯O6^i^	0.93	2.46	3.350 (3)	160
C3—H3*B*⋯O1^ii^	0.97	2.36	3.151 (3)	138
O5—H2*M*⋯N6^iii^	0.77 (3)	1.95 (3)	2.721 (2)	172 (3)
O6—H2*A*⋯O4	0.99 (4)	1.63 (4)	2.547 (3)	151 (4)

Symmetry codes: (i) 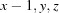; (ii) 
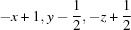
; (iii) 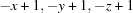.

**Fig. 1.**
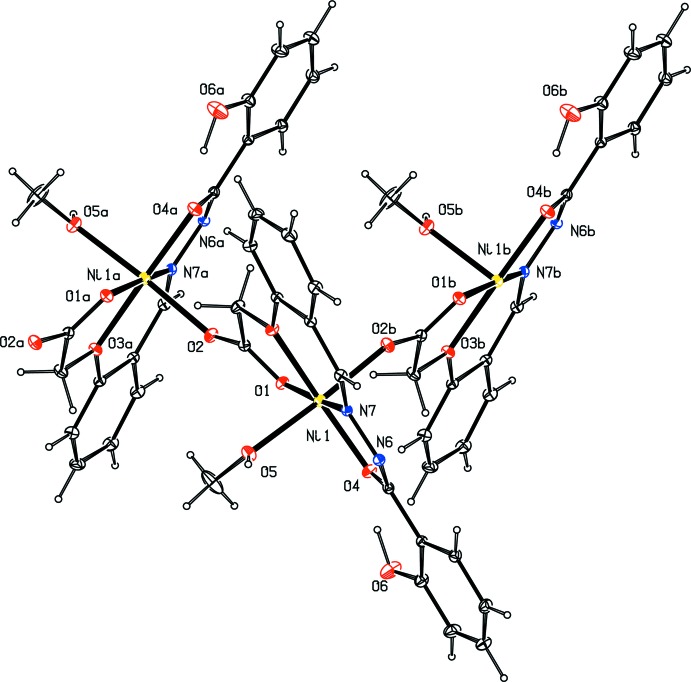
Part of the 1-D structure of the title compound with displacement ellipsoids drawn at the 30% probability level [symmetry codes: (a) -x+1, y-1/2, -z+1/2; (b) -x+1, y+1/2, -z+1/2].

**Fig. 2.**
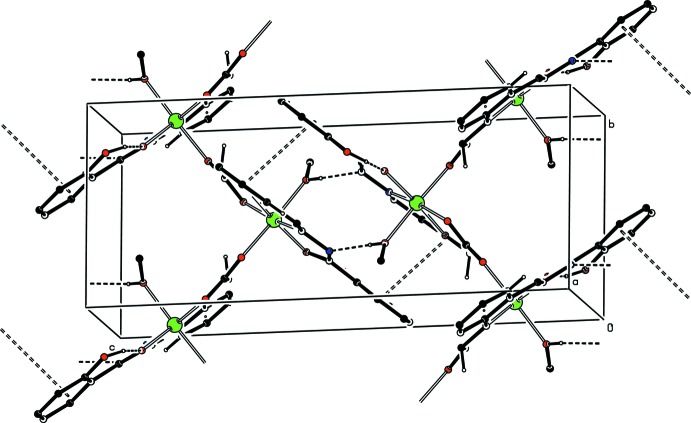
Part of the crystal structure with hydrogen bonds drawn as dashed lines. Only H atoms involved in hydrogen bonds are shown.

## supplementary crystallographic information

### 1. Comment

Nickel(II) carboxylates, especially those with nitrogen-donor ligands, have been the subject of numerous investigations (Lu *et al.*, 2010). Different coordination modes of carboxylate groups can form mononuclear and polynuclear structures. Hydrazone with carboxylate groups can also form mono- and polynuclear structures under different conditions (Wu *et al.*, 2007; Luo *et al.*, 2010). Herein we report the synthesis and crystal structure of the title compound, (I).

Part of the one-dimensional structure of (I) is shown in Fig. 1. The unique Ni^II^ ion is coordinated in a distorted octahedral environment by three O atoms and one N atom from a symmetry-unique ligand. One axial site is coordinated by a methanol solvent and a symmetry-related ligand provides an O atom from a carboxylate group to complete the coordination in the other axial site and generate a one-dimensional polymer parallel to [010]. In the crystal, O—H···O hydrogen bonds and π–π interactions, with a centroid–centroid distance of 3.693 (2) Å, form a two-dimensional network parallel to (100). In addition, weak C—H···O and C—H···N hydrogen bonds complete a three-dimensional network (Fig. 2). An intramolecular O—H···O hydrogen bond is also observed.

### 2. Experimental

The hydrazone ligand was synthesized according to the literature procedure (Luo *et al.*, 2010). Nickel(II) acetate monohydrate (1 mmol) was dissolved in methanol (15 ml), to which a solution of the ligand (2.5 mmol) in dimethylformamide (15 ml) was added. The mixture was stirred for 3 h at room temperature. A light-green solution was obtained, the solution was filtered and allowed to stand at room temperature for three weeks, where upon light-green block-shaped crystals were obtained.

### 3. Refinement

All H atoms, except for H2A, H1M and H2M were placed in idealized positions and allowed to ride on their parent atoms, with C—H = 0.93-0.97Å and U_iso_=1.2–1.5U_eq_(C). The hydroxy H2A, H1M and H2M atoms were refined independently with an isotropic displacement parameters.

### Crystal data

**Table:**

[Ni(C_16_H_12_N_2_O_5_)(CH_4_O)]	*F*(000) = 832
*M**_r_* = 403.03	*D*_x_ = 1.614 Mg m^−^^3^
Monoclinic, *P*2_1_/*c*	Mo *K*α radiation, λ = 0.71073 Å
Hall symbol: -P 2ybc	Cell parameters from 6447 reflections
*a* = 10.0877 (14) Å	θ = 2.6–27.6°
*b* = 8.1922 (10) Å	µ = 1.21 mm^−^^1^
*c* = 20.570 (2) Å	*T* = 296 K
β = 102.589 (8)°	Block, green
*V* = 1659.0 (4) Å^3^	0.20 × 0.20 × 0.15 mm
*Z* = 4	

### Data collection

**Table:**

Bruker SMART CCD diffractometer	2635 reflections with *I* > 2σ(*I*)
Radiation source: fine-focus sealed tube	*R*_int_ = 0.027
Graphite monochromator	θ_max_ = 25.0°, θ_min_ = 2.0°
φ and ω scans	*h* = −11→8
12305 measured reflections	*k* = −9→9
2921 independent reflections	*l* = −24→24

### Refinement

**Table:**

Refinement on *F*^2^	Primary atom site location: structure-invariant direct methods
Least-squares matrix: full	Secondary atom site location: difference Fourier map
*R*[*F*^2^ > 2σ(*F*^2^)] = 0.029	Hydrogen site location: inferred from neighbouring sites
*wR*(*F*^2^) = 0.073	H atoms treated by a mixture of independent and constrained refinement
*S* = 1.08	*w* = 1/[σ^2^(*F*_o_^2^) + (0.0266*P*)^2^ + 1.2361*P*] where *P* = (*F*_o_^2^ + 2*F*_c_^2^)/3
2921 reflections	(Δ/σ)_max_ = 0.001
248 parameters	Δρ_max_ = 0.35 e Å^−^^3^
0 restraints	Δρ_min_ = −0.24 e Å^−^^3^

### Special details

**Table:**

Geometry. All esds (except the esd in the dihedral angle between two l.s. planes) are estimated using the full covariance matrix. The cell esds are taken into account individually in the estimation of esds in distances, angles and torsion angles; correlations between esds in cell parameters are only used when they are defined by crystal symmetry. An approximate (isotropic) treatment of cell esds is used for estimating esds involving l.s. planes.
Refinement. Refinement of F^2^ against ALL reflections. The weighted R-factor wR and goodness of fit S are based on F^2^, conventional R-factors R are based on F, with F set to zero for negative F^2^. The threshold expression of F^2^ > 2sigma(F^2^) is used only for calculating R-factors(gt) etc. and is not relevant to the choice of reflections for refinement. R-factors based on F^2^ are statistically about twice as large as those based on F, and R- factors based on ALL data will be even larger.

### Fractional atomic coordinates and isotropic or equivalent isotropic displacement parameters (Å^2^)

**Table:**

	*x*	*y*	*z*	*U*_iso_*/*U*_eq_	
C1	0.6935 (4)	0.2217 (5)	0.41187 (18)	0.0885 (14)	
H1A	0.6769	0.1599	0.3712	0.133*	
H1B	0.7039	0.1485	0.4491	0.133*	
H1C	0.7749	0.2848	0.4154	0.133*	
C2	0.5242 (2)	0.3099 (3)	0.24797 (11)	0.0298 (5)	
C3	0.3753 (2)	0.2907 (3)	0.25087 (12)	0.0378 (6)	
H3A	0.3179	0.3249	0.2089	0.045*	
H3B	0.3560	0.1770	0.2581	0.045*	
C4	0.1090 (2)	0.3233 (3)	0.26828 (12)	0.0394 (6)	
H4	0.1274	0.2588	0.2341	0.047*	
C5	−0.0237 (3)	0.3400 (4)	0.27576 (13)	0.0453 (7)	
H5	−0.0936	0.2867	0.2465	0.054*	
C6	−0.0525 (3)	0.4341 (4)	0.32578 (14)	0.0450 (7)	
H6	−0.1418	0.4470	0.3301	0.054*	
C7	0.0517 (3)	0.5092 (3)	0.36958 (14)	0.0401 (6)	
H7	0.0315	0.5713	0.4040	0.048*	
C8	0.1880 (2)	0.4959 (3)	0.36431 (11)	0.0299 (5)	
C9	0.2136 (2)	0.4015 (3)	0.31103 (11)	0.0290 (5)	
C10	0.2849 (2)	0.5833 (3)	0.41492 (11)	0.0312 (5)	
C11	0.6105 (2)	0.7209 (3)	0.45898 (11)	0.0293 (5)	
C12	0.7036 (2)	0.8173 (3)	0.50982 (11)	0.0299 (5)	
C13	0.6566 (3)	0.9116 (3)	0.55665 (11)	0.0365 (6)	
H13	0.5638	0.9154	0.5552	0.044*	
C14	0.7437 (3)	0.9989 (3)	0.60491 (13)	0.0444 (7)	
H14	0.7106	1.0602	0.6360	0.053*	
C15	0.8805 (3)	0.9942 (4)	0.60641 (14)	0.0527 (8)	
H15	0.9403	1.0524	0.6390	0.063*	
C16	0.9299 (3)	0.9054 (4)	0.56088 (15)	0.0543 (8)	
H16	1.0228	0.9044	0.5625	0.065*	
C17	0.8429 (3)	0.8170 (3)	0.51235 (13)	0.0418 (6)	
H2A	0.818 (4)	0.691 (5)	0.4351 (19)	0.106 (14)*	
H1M	0.251 (2)	0.630 (3)	0.4492 (12)	0.033 (6)*	
H2M	0.558 (3)	0.323 (4)	0.4441 (16)	0.064 (11)*	
N6	0.48640 (18)	0.6936 (2)	0.46842 (9)	0.0285 (4)	
N7	0.41149 (18)	0.6014 (2)	0.41628 (8)	0.0261 (4)	
Ni1	0.51359 (3)	0.52553 (4)	0.351123 (13)	0.02633 (11)	
O1	0.59405 (15)	0.4187 (2)	0.28177 (7)	0.0318 (4)	
O2	0.56805 (17)	0.2161 (2)	0.21012 (8)	0.0378 (4)	
O3	0.34651 (15)	0.3882 (2)	0.30411 (7)	0.0320 (4)	
O4	0.65470 (16)	0.6669 (2)	0.40854 (8)	0.0358 (4)	
O5	0.58543 (18)	0.3247 (2)	0.41172 (9)	0.0412 (4)	
O6	0.8977 (2)	0.7297 (3)	0.46879 (12)	0.0714 (7)	

### Atomic displacement parameters (Å^2^)

**Table:**

	*U*^11^	*U*^22^	*U*^33^	*U*^12^	*U*^13^	*U*^23^
C1	0.102 (3)	0.104 (3)	0.076 (2)	0.067 (2)	0.056 (2)	0.044 (2)
C2	0.0348 (13)	0.0315 (13)	0.0249 (11)	0.0008 (10)	0.0104 (10)	0.0013 (10)
C3	0.0346 (13)	0.0427 (15)	0.0385 (13)	−0.0032 (11)	0.0131 (11)	−0.0141 (11)
C4	0.0355 (13)	0.0452 (16)	0.0368 (13)	−0.0021 (11)	0.0064 (11)	−0.0017 (11)
C5	0.0317 (13)	0.0550 (18)	0.0453 (15)	−0.0085 (12)	0.0002 (11)	0.0051 (13)
C6	0.0256 (13)	0.0551 (18)	0.0558 (17)	0.0022 (12)	0.0124 (12)	0.0107 (14)
C7	0.0320 (13)	0.0476 (16)	0.0436 (14)	0.0047 (11)	0.0145 (11)	0.0018 (12)
C8	0.0267 (12)	0.0330 (13)	0.0310 (12)	0.0014 (9)	0.0083 (10)	0.0049 (10)
C9	0.0241 (11)	0.0327 (13)	0.0308 (12)	0.0008 (9)	0.0076 (9)	0.0045 (10)
C10	0.0309 (12)	0.0366 (14)	0.0292 (12)	0.0044 (10)	0.0134 (10)	−0.0014 (10)
C11	0.0345 (12)	0.0277 (12)	0.0267 (11)	0.0017 (10)	0.0087 (10)	0.0021 (9)
C12	0.0322 (12)	0.0285 (13)	0.0285 (11)	0.0000 (10)	0.0058 (9)	0.0009 (10)
C13	0.0411 (14)	0.0351 (14)	0.0344 (13)	−0.0014 (11)	0.0105 (11)	−0.0018 (11)
C14	0.0595 (18)	0.0386 (15)	0.0349 (14)	−0.0015 (13)	0.0097 (13)	−0.0081 (11)
C15	0.0562 (18)	0.0518 (18)	0.0439 (16)	−0.0095 (14)	−0.0028 (14)	−0.0122 (13)
C16	0.0345 (14)	0.066 (2)	0.0589 (18)	−0.0033 (14)	0.0015 (13)	−0.0128 (16)
C17	0.0371 (14)	0.0431 (16)	0.0460 (15)	−0.0007 (12)	0.0107 (12)	−0.0088 (12)
N6	0.0313 (10)	0.0305 (11)	0.0245 (9)	0.0006 (8)	0.0080 (8)	−0.0017 (8)
N7	0.0302 (10)	0.0261 (10)	0.0231 (9)	0.0000 (8)	0.0084 (8)	0.0016 (8)
Ni1	0.02821 (18)	0.02915 (19)	0.02373 (16)	−0.00093 (12)	0.01027 (12)	−0.00196 (11)
O1	0.0329 (8)	0.0358 (10)	0.0293 (8)	−0.0050 (7)	0.0127 (7)	−0.0060 (7)
O2	0.0417 (10)	0.0401 (10)	0.0360 (9)	−0.0043 (8)	0.0177 (8)	−0.0123 (8)
O3	0.0270 (8)	0.0386 (10)	0.0325 (8)	−0.0024 (7)	0.0111 (7)	−0.0104 (7)
O4	0.0343 (9)	0.0456 (10)	0.0310 (8)	−0.0075 (8)	0.0149 (7)	−0.0108 (8)
O5	0.0476 (11)	0.0462 (11)	0.0352 (10)	0.0138 (8)	0.0206 (9)	0.0092 (8)
O6	0.0373 (11)	0.0965 (19)	0.0834 (16)	−0.0034 (11)	0.0199 (11)	−0.0473 (14)

### Geometric parameters (Å, º)

**Table:**

C1—O5	1.378 (3)	C11—O4	1.293 (3)
C1—H1A	0.9600	C11—N6	1.327 (3)
C1—H1B	0.9600	C11—C12	1.473 (3)
C1—H1C	0.9600	C12—C17	1.395 (3)
C2—O2	1.242 (3)	C12—C13	1.396 (3)
C2—O1	1.249 (3)	C13—C14	1.374 (3)
C2—C3	1.524 (3)	C13—H13	0.9300
C3—O3	1.435 (3)	C14—C15	1.375 (4)
C3—H3A	0.9700	C14—H14	0.9300
C3—H3B	0.9700	C15—C16	1.363 (4)
C4—C9	1.375 (3)	C15—H15	0.9300
C4—C5	1.387 (3)	C16—C17	1.382 (4)
C4—H4	0.9300	C16—H16	0.9300
C5—C6	1.367 (4)	C17—O6	1.355 (3)
C5—H5	0.9300	N6—N7	1.393 (2)
C6—C7	1.373 (4)	N7—Ni1	1.9608 (17)
C6—H6	0.9300	Ni1—O1	1.9914 (15)
C7—C8	1.407 (3)	Ni1—O4	2.0076 (16)
C7—H7	0.9300	Ni1—O2^i^	2.0615 (16)
C8—C9	1.410 (3)	Ni1—O3	2.0810 (15)
C8—C10	1.452 (3)	Ni1—O5	2.0954 (18)
C9—O3	1.384 (3)	O2—Ni1^ii^	2.0615 (16)
C10—N7	1.280 (3)	O5—H2M	0.77 (3)
C10—H1M	0.93 (2)	O6—H2A	0.99 (4)
			
O5—C1—H1A	109.5	C14—C13—H13	119.1
O5—C1—H1B	109.5	C12—C13—H13	119.1
H1A—C1—H1B	109.5	C15—C14—C13	118.8 (3)
O5—C1—H1C	109.5	C15—C14—H14	120.6
H1A—C1—H1C	109.5	C13—C14—H14	120.6
H1B—C1—H1C	109.5	C16—C15—C14	121.0 (3)
O2—C2—O1	123.7 (2)	C16—C15—H15	119.5
O2—C2—C3	116.7 (2)	C14—C15—H15	119.5
O1—C2—C3	119.5 (2)	C15—C16—C17	120.4 (3)
O3—C3—C2	109.71 (18)	C15—C16—H16	119.8
O3—C3—H3A	109.7	C17—C16—H16	119.8
C2—C3—H3A	109.7	O6—C17—C16	117.9 (2)
O3—C3—H3B	109.7	O6—C17—C12	122.0 (2)
C2—C3—H3B	109.7	C16—C17—C12	120.1 (2)
H3A—C3—H3B	108.2	C11—N6—N7	110.46 (17)
C9—C4—C5	120.5 (2)	C10—N7—N6	116.80 (18)
C9—C4—H4	119.8	C10—N7—Ni1	128.23 (16)
C5—C4—H4	119.8	N6—N7—Ni1	114.88 (13)
C6—C5—C4	120.5 (2)	N7—Ni1—O1	170.34 (7)
C6—C5—H5	119.7	N7—Ni1—O4	79.97 (7)
C4—C5—H5	119.7	O1—Ni1—O4	109.13 (6)
C5—C6—C7	119.3 (2)	N7—Ni1—O2^i^	88.76 (7)
C5—C6—H6	120.4	O1—Ni1—O2^i^	93.79 (7)
C7—C6—H6	120.4	O4—Ni1—O2^i^	93.45 (7)
C6—C7—C8	122.4 (3)	N7—Ni1—O3	89.77 (7)
C6—C7—H7	118.8	O1—Ni1—O3	81.07 (6)
C8—C7—H7	118.8	O4—Ni1—O3	169.71 (6)
C7—C8—C9	116.7 (2)	O2^i^—Ni1—O3	87.14 (7)
C7—C8—C10	115.0 (2)	N7—Ni1—O5	90.41 (7)
C9—C8—C10	128.2 (2)	O1—Ni1—O5	86.50 (7)
C4—C9—O3	121.5 (2)	O4—Ni1—O5	89.66 (7)
C4—C9—C8	120.5 (2)	O2^i^—Ni1—O5	176.60 (7)
O3—C9—C8	117.95 (19)	O3—Ni1—O5	89.55 (7)
N7—C10—C8	125.8 (2)	C2—O1—Ni1	116.33 (14)
N7—C10—H1M	117.7 (14)	C2—O2—Ni1^ii^	134.59 (15)
C8—C10—H1M	116.4 (14)	C9—O3—C3	118.95 (17)
O4—C11—N6	124.1 (2)	C9—O3—Ni1	127.93 (14)
O4—C11—C12	118.3 (2)	C3—O3—Ni1	112.18 (13)
N6—C11—C12	117.53 (19)	C11—O4—Ni1	110.17 (14)
C17—C12—C13	117.8 (2)	C1—O5—Ni1	130.77 (18)
C17—C12—C11	120.4 (2)	C1—O5—H2M	115 (2)
C13—C12—C11	121.8 (2)	Ni1—O5—H2M	113 (2)
C14—C13—C12	121.8 (2)	C17—O6—H2A	104 (2)
			
O2—C2—C3—O3	170.7 (2)	C10—N7—Ni1—O3	−10.1 (2)
O1—C2—C3—O3	−10.8 (3)	N6—N7—Ni1—O3	173.58 (14)
C9—C4—C5—C6	−0.1 (4)	C10—N7—Ni1—O5	−99.7 (2)
C4—C5—C6—C7	−1.4 (4)	N6—N7—Ni1—O5	84.03 (14)
C5—C6—C7—C8	1.3 (4)	O2—C2—O1—Ni1	−168.85 (18)
C6—C7—C8—C9	0.4 (4)	C3—C2—O1—Ni1	12.7 (3)
C6—C7—C8—C10	−180.0 (2)	O4—Ni1—O1—C2	170.61 (15)
C5—C4—C9—O3	−179.3 (2)	O2^i^—Ni1—O1—C2	−94.41 (16)
C5—C4—C9—C8	1.9 (4)	O3—Ni1—O1—C2	−7.90 (16)
C7—C8—C9—C4	−2.0 (3)	O5—Ni1—O1—C2	82.19 (17)
C10—C8—C9—C4	178.5 (2)	O1—C2—O2—Ni1^ii^	179.20 (16)
C7—C8—C9—O3	179.1 (2)	C3—C2—O2—Ni1^ii^	−2.3 (3)
C10—C8—C9—O3	−0.4 (4)	C4—C9—O3—C3	0.2 (3)
C7—C8—C10—N7	−172.9 (2)	C8—C9—O3—C3	179.0 (2)
C9—C8—C10—N7	6.6 (4)	C4—C9—O3—Ni1	168.12 (17)
O4—C11—C12—C17	−15.3 (3)	C8—C9—O3—Ni1	−13.0 (3)
N6—C11—C12—C17	164.0 (2)	C2—C3—O3—C9	173.36 (19)
O4—C11—C12—C13	164.5 (2)	C2—C3—O3—Ni1	3.6 (2)
N6—C11—C12—C13	−16.1 (3)	N7—Ni1—O3—C9	16.00 (18)
C17—C12—C13—C14	−1.5 (4)	O1—Ni1—O3—C9	−167.07 (18)
C11—C12—C13—C14	178.7 (2)	O4—Ni1—O3—C9	20.8 (5)
C12—C13—C14—C15	0.7 (4)	O2^i^—Ni1—O3—C9	−72.77 (17)
C13—C14—C15—C16	0.3 (4)	O5—Ni1—O3—C9	106.41 (18)
C14—C15—C16—C17	−0.5 (5)	N7—Ni1—O3—C3	−175.38 (16)
C15—C16—C17—O6	−179.4 (3)	O1—Ni1—O3—C3	1.56 (15)
C15—C16—C17—C12	−0.3 (5)	O4—Ni1—O3—C3	−170.6 (4)
C13—C12—C17—O6	−179.7 (3)	O2^i^—Ni1—O3—C3	95.85 (16)
C11—C12—C17—O6	0.2 (4)	O5—Ni1—O3—C3	−84.97 (16)
C13—C12—C17—C16	1.3 (4)	N6—C11—O4—Ni1	−3.8 (3)
C11—C12—C17—C16	−178.9 (2)	C12—C11—O4—Ni1	175.49 (16)
O4—C11—N6—N7	−0.7 (3)	N7—Ni1—O4—C11	4.87 (15)
C12—C11—N6—N7	180.00 (18)	O1—Ni1—O4—C11	−171.78 (14)
C8—C10—N7—N6	178.3 (2)	O2^i^—Ni1—O4—C11	93.00 (15)
C8—C10—N7—Ni1	2.1 (4)	O3—Ni1—O4—C11	0.0 (5)
C11—N6—N7—C10	−171.6 (2)	O5—Ni1—O4—C11	−85.61 (15)
C11—N6—N7—Ni1	5.1 (2)	N7—Ni1—O5—C1	−162.2 (3)
C10—N7—Ni1—O4	170.7 (2)	O1—Ni1—O5—C1	27.0 (3)
N6—N7—Ni1—O4	−5.55 (14)	O4—Ni1—O5—C1	−82.2 (3)
C10—N7—Ni1—O2^i^	77.0 (2)	O3—Ni1—O5—C1	108.1 (3)
N6—N7—Ni1—O2^i^	−99.27 (14)		

Symmetry codes: (i) −*x*+1, *y*+1/2, −*z*+1/2; (ii) −*x*+1, *y*−1/2, −*z*+1/2.

### Hydrogen-bond geometry (Å, º)

**Table:**

*D*—H···*A*	*D*—H	H···*A*	*D*···*A*	*D*—H···*A*
C7—H7···O6^iii^	0.93	2.46	3.350 (3)	160
C3—H3*B*···O1^ii^	0.97	2.36	3.151 (3)	138
O5—H2*M*···N6^iv^	0.77 (3)	1.95 (3)	2.721 (2)	172 (3)
O6—H2*A*···O4	0.99 (4)	1.63 (4)	2.547 (3)	151 (4)

Symmetry codes: (ii) −*x*+1, *y*−1/2, −*z*+1/2; (iii) *x*−1, *y*, *z*; (iv) −*x*+1, −*y*+1, −*z*+1.
